# DNA Damage and Repair in Pulmonary Arterial Hypertension

**DOI:** 10.3390/genes11101224

**Published:** 2020-10-19

**Authors:** Samantha Sharma, Micheala A. Aldred

**Affiliations:** Division of Pulmonary, Critical Care, Sleep & Occupational Medicine, Department of Medicine, Indiana University School of Medicine, Indianapolis, IN 46202, USA; samshar@iu.edu

**Keywords:** pulmonary arterial hypertension, endothelial cells, smooth muscle cells, DNA damage, DNA repair

## Abstract

Pulmonary arterial hypertension (PAH) is a complex multifactorial disease with both genetic and environmental dynamics contributing to disease progression. Over the last decade, several studies have demonstrated the presence of genomic instability and increased levels of DNA damage in PAH lung vascular cells, which contribute to their pathogenic apoptosis-resistant and proliferating characteristics. In addition, the dysregulated DNA damage response pathways have been indicated as causal factors for the presence of persistent DNA damage. To understand the significant implications of DNA damage and repair in PAH pathogenesis, the current review summarizes the recent advances made in this field. This includes an overview of the observed DNA damage in the nuclear and mitochondrial genome of PAH patients. Next, the irregularities observed in various DNA damage response pathways and their role in accumulating DNA damage, escaping apoptosis, and proliferation under a DNA damaging environment are discussed. Although the current literature establishes the pertinence of DNA damage in PAH, additional studies are required to understand the temporal sequence of the above-mentioned events. Further, an exploration of different types of DNA damage in conjunction with associated impaired DNA damage response in PAH will potentially stimulate early diagnosis of the disease and development of novel therapeutic strategies.

## 1. Introduction

Pulmonary arterial hypertension (PAH) is a potentially fatal vascular disease of enigmatic origin [1]. Following recent revision at the 6th World Symposium on Pulmonary Hypertension, it is now defined as a mean pulmonary artery pressure greater than 20 mmHg at rest, pulmonary artery wedge pressure of ≤15 mmHg, and pulmonary vascular resistance ≥3 Wood Units [2]. It is sub-divided into the following categories: heritable PAH (HPAH) with a known genetic mutation and/or a family history; idiopathic (IPAH) with no known cause; associated PAH (APAH) that occurs in concert with a predisposing condition, such as connective tissue disorder (CTD), congenital heart disease (CHD), or ingestion of certain drugs and toxins; and PAH with overt features of venous/capillary involvement (PVOD/PCH) [2]. PAH is characterized by remodeling of the pre-capillary arterioles that is directed by endothelial cell (EC) dysfunction, abnormal smooth muscle cell (SMC) proliferation, and proinflammatory cytokines [2,3,4,5,6]. These vascular abnormalities lead to progressive obliteration of the proximal pulmonary arterioles and pruning of the distal microvessels, thus increasing resistance in the pulmonary artery (PA) and ultimately leading to right heart failure [1,4,5]. Current therapies provide symptomatic relief; however, the 5-year survival rate of PAH remains below 60%, with lung transplantation being the only curative option [4,7,8,9]. A better understanding of the early molecular events in PAH pathogenesis would potentially aid earlier diagnosis and the development of targeted therapies.

PAH is a complex multifactorial disease with both genetic and environmental factors contributing to the pathogenesis. HPAH is inherited as an autosomal dominant trait with reduced penetrance. Mutations in the bone morphogenetic protein receptor II gene (*BMPR2*) are by far the predominant cause [10,11,12], but recent whole-exome and genome sequencing efforts have identified numerous other genes, including several other members of the BMP pathway [12,13,14,15,16,17]. However, the low penetrance of these mutations suggests that additional factors are required to trigger the disease. Such events may include other aberrations within the genome [18,19], and/or environmental stressors such as hypoxia or inflammation [5,20,21,22,23,24,25].

Over the last two decades, several reports have highlighted a role for DNA damage in PAH pathogenesis. The primary cause of this DNA damage remains uncertain. Depending on the type of DNA damage, the cell manifests a specific DNA damage response (DDR) cascade that is responsible for the recognition, signaling, and repair process [26]. DDR is accompanied by increased nucleotide synthesis, transcriptional and epigenetic changes, and metabolic amendments via higher consumption of glucose [26,27]. These alterations disrupt the cellular homeostasis and prompt the cell’s signaling network to determine the cell’s survival fate. In this review, we discuss the hallmark discoveries surrounding DNA damage and associated repair pathways to understand their potential roles in PAH vascular remodeling.

## 2. Background: DNA Damage and Response Pathways

**Figure 1:**
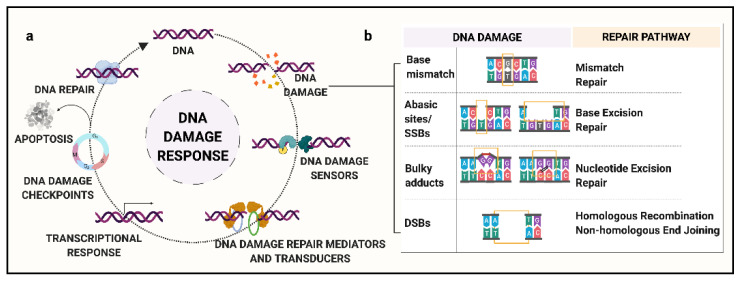
Pictorial representation of (**a**) DNA damage response and (**b**) types of DNA damage and their repair pathways. (**a**) DNA damage response includes the recognition of DNA damage by DNA damage sensors, recruitment of DNA damage repair mediators and transducers, modulation of transcriptional response, activation of DNA damage checkpoint kinases, and lastly, restoration of DNA duplex or apoptosis signaling if the damage is unrepairable. (**b**) Types of DNA damage include base mismatches repaired via mismatch repair pathway, abasic site or SSBs repaired via base excision repair, bulky adducts repaired via nucleotide excision repair, and DSBs repaired via homologous recombination or non-homologous end joining. DNA, deoxyribonucleic acid; SSBs, single-stranded DNA breaks; DSBs, double-stranded DNA breaks. Created with BioRender.com.

Continuous exposure of genomic DNA to cellular metabolites and exogenous agents can damage the structural integrity of DNA. These alterations can range from a single base to complex structural changes. Based on the type of DNA damage, relevant DDR pathways are activated [27]. These responses work towards (a) restoration of the DNA duplex, (b) activation of DNA damage checkpoint kinase 1 and 2 (CHK1 and CHK2) to prevent transmission of damaged DNA, (c) transcriptional alterations to maintain cellular health, and (d) apoptosis signaling if the damage is unrepairable (Figure 1a) [26].

DNA damage includes single-stranded DNA breaks (SSBs), abasic sites, modified bases, double-stranded DNA breaks (DSBs), and inter- and intra-strand crosslinks (Figure 1b) [26]. Abasic sites, SSBs, and DSBs fall into the category of DNA backbone damage, which is the most frequent type of DNA damage [26]. Abasic or AP sites (apurinic/apyrimidinic site) are characterized by the absence of a single base from the DNA backbone. SSBs are the type of DNA damage that affect only one strand of the duplex DNA, characterized by gaps in the range of up to 30 nucleotides. DSBs, or damage to both the strands of DNA, are the most lethal to DNA integrity, with the capability to generate large chromosomal aberrations. Certain chemical agents are known to generate intra- and inter-strand DNA-DNA crosslinks that can halt transcription or replication machinery [27].

DDR initiates DNA repair pathways that replace potentially damaged sites with newly synthesized DNA via base excision or recombination mechanism [26]. In humans, based on the type of DNA damage, the DNA repair mechanism can be classified into five major types (Figure 1b):

(a) Mismatch Repair (MMR): MMR is responsible for the recognition and repair of base mismatches. Base mismatches can arise as a result of covalent or non-covalent structural changes, or due to insertion/deletions resulting from replication errors or recombination [28]. For example, methylated guanine base, O6MeGua, has a high frequency of pairing to thymine (T), activating MMR to excise the mismatched T residue. Loss of MMR can lead to a significant increase in spontaneous mutations. Major known genes in the MMR pathway include *MGMT*, *MSH6*, and *MLH3*.
(b) Base Excision Repair (BER): This repair process is governed by DNA glycosylases along with endonucleases that recognize and eliminate the modified or damaged bases, such as oxidized, reduced, alkylated or deaminated bases, to generate an abasic site [29]. For example, in humans, 8-oxoguanine glycosylase-1 (OGG1) recognizes and removes the oxidatively modified guanine base, 8-oxoGuanine (8-oxoG) via incision of the 3′-phosphodiester bond. Following this step, the apurinic/apyrimidinic endodeoxyribonuclease 1 (APEX1) cleaves the 5′-bond generating a 1-nt abasic site [30]. Major genes of the BER pathway include *MBD4*, *OGG1*, *MUTYH*, and *NEIL1*.
(c) Nucleotide Excision Repair (NER): Unlike BER, NER involves a complex of enzymes that work in coordination to recognize SSBs and remove bulky lesions [31]. Briefly, the steps include recognition of the damaged site, a dual incision at extreme ends of the lesion, elimination of damaged oligomer, and new base synthesis followed by ligation [32]. Major known NER genes include *XPC*, *XPA*, and *ERCC1-5*.
(d) Homologous Recombination (HR): As compared to the excision repair pathways, HR is a far more complex phenomenon. HR involves multiple-step processing of DSBs by several different proteins with specific functions [33]. The key characteristic of HR is that it uses a homologous duplex template to retrieve the lost information. It is a complex phenomenon, with the potential for incorrect template usage that can lead to gene conversion. Major genes involved in the HR pathway include *RAD51*, *BRCA1*, *BRCA2*, and the Mre11/Rad50/NBS1 complex [34].
(e) Non-Homologous End Joining (NHEJ): Similar to HR, NHEJ involves multiple-step repair processing of DSBs. In this mechanism, the two ends of DSBs are stabilized by DNA-protein kinases and ligated together [35]. It is believed to be the main repair pathway for DSBs induced by ionizing radiation. Major proteins implicated in NHEJ include KU70/80 heterodimer and XRCC4 [36,37]. A lack of specific recognition criteria for the ligated ends can lead to erroneous joining of non-contiguous DNA sequences, giving rise to structural rearrangements.

After the initiation of the DNA repair pathway, the cell deploys DNA repair checkpoint kinases, CHK1 and CHK2, that delay or inhibit the DDR-associated cell cycle progression. Checkpoint kinases arrest cell cycle until the repair process is complete, avoiding transmission of damaged DNA to the daughter cells [38]. This step is a significant player in DDR as it halts the replication of damaged DNA and ensures the transmission of intact healthy DNA. If the DNA damage cannot be repaired, checkpoint kinases initiate apoptosis signaling, leading to cell death. Owing to their pertinence in DDR, checkpoint-specific damage sensors, ataxia telangiectasia mutated (ATM) and Ataxia Telangiectasia and Rad3-Related Protein (ATR), have received the highest recognition. Checkpoint kinases also regulate the biochemical pathways that guard different steps of the cell cycle and hence play a vital role in DDR.

## 3. DNA Damage and Genetic Instability in PAH

In 1998, it was first discovered that pulmonary artery ECs (PAECs) within the majority of plexiform lesions microdissected from IPAH lung tissues were monoclonal, suggesting that each lesion arose from the proliferation of a single EC [39,40]. Similar results were obtained in lungs from patients with appetite suppressant-associated PH, whereas lesions from the lungs of patients with CHD-PAH or CTD-PAH were polyclonal [39,40]. Thereafter, subsequent studies focused on exploring the mechanisms that confer such ECs with a unique selective growth advantage. The similarity of PAECs monoclonal expansion with neoplasia anticipated the role of an unstable genome in PAECs that favors the disruption of the apoptotic signals [41]. Soon after, the first paper to report microsatellite instability within the PAECs from plexiform lesions of PAH patients affirmed this hypothesis [42]. PAH patients reportedly had microsatellite instabilities within the transforming growth factor-β receptor II (*TGFBR2*) and BCL-2 associated X, apoptosis regulator (*BAX*) genes, known to regulate cell proliferation and apoptosis, respectively. Further, mutations within these microsatellite sites resulted in truncated proteins, thereby producing lower levels of the functional proteins. Microsatellite instability, or a condition of genetic hypermutability, results from the loss of functional DNA mismatch repair process. Hence, these quasi-neoplastic PAECs bearing unstable microsatellite mutations suggested the relevance of DNA damage and DNA repair regulation in the pathophysiology of PAH.

Subsequent to the identification of *BMPR2* mutations in heritable PAH, Machado et al. tested the hypothesis that this gene might follow a classical two-hit tumor suppressor model, with somatic loss of the wildtype allele in lung vascular lesions triggering disease onset [43]. However, careful microdissection and genetic analysis disproved this hypothesis, suggesting that if somatic mutations existed, they lie elsewhere in the genome. Our lab then performed a genome-wide microarray copy number analysis in cultured PAECs and PASMCs isolated from the lungs of patients with idiopathic, heritable, and associated forms of PAH [44]. Chromosomal deletions were identified in PAECs from five of the nine cases studied and validated in the uncultured lung tissue. These aberrations were not detectable in paired DNA samples from blood or other lung cell types, confirming that they were somatic events. In the same study, a second-hit mechanism became evident when a patient with a germline *BMPR2* mutation was found to harbor somatic loss of one copy of chromosome-13, which deleted one of the BMP signaling transducer genes, SMAD family member 9 (*SMAD9*) [44]. Subsequently, a detailed analysis of an interstitial chromosome-13 deletion in PAEC from an APAH patient revealed dysregulated BMP signaling, similar to that seen in HPAH cells bearing a germline *SMAD9* mutation [45]. In contrast, PASMC from the same patient did not carry the deletion and showed normal BMP signaling. Validation of chromosomal abnormalities in a larger sample size confirmed a significant excess of copy number changes in PAH-PAECs (30.9%) as compared to the control-PAECs (5.3%), whereas the frequency in PASMCs did not differ between patients and controls [46].

These studies identified a surprisingly high level of DNA damage in the lung tissues of PAH patients. However, its relevance to PAH pathophysiology was yet to be understood. Is increased DNA damage an intrinsic property of a cell acting as a disease driver, or simply an end-stage consequence in PAH pathogenesis? To answer this question, we directly measured DNA damage in PAECs and blood cells using micronucleus assay and immunocytochemistry for phosphorylated Histone H2a Family Member X (γH2AX), a marker of double-strand break repair. PAH cells showed higher levels of DNA damage than controls, both in PAEC and in blood cells [46]. Reactive oxygen species (ROS) levels were also elevated in PAH cells. Treatment with antioxidants reduced the level of DNA damage to a similar baseline as control cells, suggesting that excess DNA damage in PAH cells may be due, at least in part, to oxidative stress. Notably, similar results were shown in blood cells from healthy first-degree relatives of PAH patients, suggesting there may be an intrinsic genetic or epigenetic basis [46].

Overall, these studies suggested ROS and DNA damage as biomarkers for PAH susceptibility across multiple PAH sub-groups, including IPAH, HPAH, and APAH. This is supported by evidence of increased DNA damage in PAECs from amphetamine-PAH lungs [47], as well as pulmonary microvascular ECs (PMVECs) from IPAH patients [48]. Furthermore, although PASMCs did not show significant evidence of chromosomal abnormalities [46], they do also exhibit higher levels of DNA damage than control cells [49].

## 4. Role of Mutagens and Environmental Modifiers

With the cellular environment as a key factor, several studies established low oxygen levels, or hypoxia, as a powerful stimulant for pathological conditions [21,24]. With relevance to DNA damage, hypoxia is reported to downregulate a cell’s DNA repair machinery, leading to an increased prevalence of genomic damage [50,51,52,53]. Amphetamines, a potent synthetic neuro-stimulant, reportedly have vasoconstrictive and mutagenic properties in vascular cells [54]. Further, oxidant injury (caused by a hypoxic environment) enhances their neurotoxic effects, proposing amphetamines as a potent trigger for vasculopathy [55]. It has been reported that people exposed to amphetamine have a 3-fold higher risk factor for the development of PAH [54]. To understand the mechanism of amphetamine-associated PAH, Chen and colleagues performed an elaborate translational study in PAECs from drug and toxin-induced PAH patients [47]. In addition to elevated levels of baseline DNA damage, PAECs derived from amphetamine-PAH lungs were also more susceptible to genotoxins as compared to the controls. Further, the genotoxic effect of doxorubicin was exaggerated under hypoxic conditions and persisted even after recovery under normoxic conditions. In vivo, animals treated with amphetamines demonstrated increased DNA damage, but no significant change in hemodynamics was observed, supporting the involvement of additional factors causing the pathogenic vulnerability in the vascular cells [47].

Other groups have also reported an augmented susceptibility to mutagens in PAH cells, including etoposide, bleomycin, and hydroxyurea [46,48]. Topoisomerase-II binding protein 1 (TOPBP1) plays a role in the rescue of stalled replication forks and checkpoint control, binding both dsDNA and ssDNA breaks. It is downregulated in PMVECs from IPAH patients, which showed evidence of increased DNA damage and apoptosis [48]. Common single-nucleotide variants in the *TOPBP1* gene modified the susceptibility of normal PMVECs to hydroxyurea, and it was proposed as a novel gene in IPAH [48]. However, this has not been validated in a recent large genome-wide association study [56].

**Figure 2:**
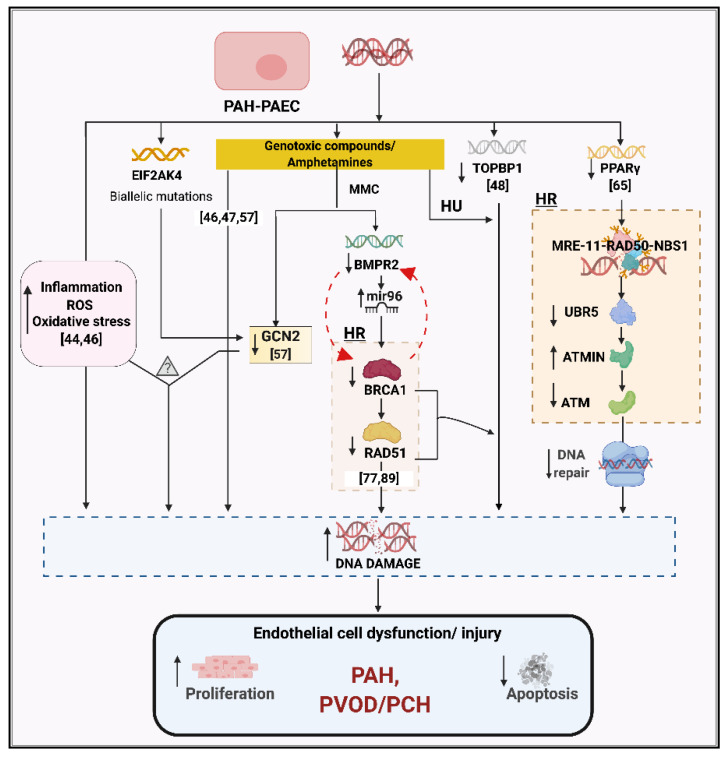
Dysregulated DNA damage response genes involved in pro-proliferative and apoptosis resistance characteristics of PAH-PAECs. Increased inflammation, ROS, oxidative stress, and susceptibility to genotoxic compounds are associated with increased DNA damage in PAH-PAECs. Reduced expression of PPARγ and BMPR2 in PAH-PAECs downregulates the downstream HR DNA repair genes, ATM and RAD51, respectively. The BMPR2-BRCA1-RAD51 pathway is also dysregulated in presence of MMC, a genotoxin. MMC is also proposed to trigger pulmonary vascular dysfunction associated with DNA damage signaling via reduced GCN2 expression, leading to PVOD. A similar mechanism may contribute to familial PVOD/PCH, where GCN2 is inactivated by biallelic *EIF2AK4* mutations. Similarly, reduced expression of TOPBP1, a DNA repair gene, in PAH-PAECs is reported to associate with PAH pathogenesis. These factors cause increased DNA damage, leading to endothelial cell dysfunction/injury in PAH and PVOD/PCH. The question mark symbol represents that the exact mechanism of this pathway in inducing the disease is still uncertain. DNA, Deoxyribonucleic acid; ROS, Reactive oxygen species; EIF2AK4, Eukaryotic translation initiation factor 2α kinase 4; GCN2, eIF-2α kinase GCN2; MMC, Mitomycin-C; BMPR2, bone morphogenetic protein receptor type 2; miR96, microRNA-96; HR, Homologous recombination; BRCA1, Breast and ovarian cancer susceptibility protein 1; RAD51, RAD51 recombinase; HU, Hydroxyurea; TopBP1, DNA Topoisomerase II Binding Protein 1; PPARγ, Peroxisome proliferator-activated receptor γ; MRE11, Double-strand break repair protein MRE11; RAD50, RAD50 double-strand break repair protein; NBS1, Nibrin; UBR5, Ubiquitin protein ligase e3 component n-recognin 5; ATMIN, ATM interacting protein; ATM, Ataxia telangiectasia mutated; PAH, Pulmonary arterial hypertension; PVOD, Pulmonary veno-occlusive disease; PCH, Pulmonary capillary hemangiomatosis. The numbers represented in square brackets cite the reference for that study. Created with BioRender.com.

The most direct evidence that mutagens can precipitate the development of pulmonary hypertension comes from pulmonary veno-occlusive disease (PVOD), a rare form of PH with an especially poor prognosis. Perros and colleagues reported that cancer patients undergoing treatment with mitomycin-C (MMC) had a significantly higher annual incidence of PVOD (3.9 out of 1000) as compared to the general population (0.5 per million) [57]. In vivo treatment of rats with MMC induced severe pulmonary vascular resistance and right ventricular (RV) hypertrophy accompanied by vascular remodeling and EC proliferation in the capillary bed. At the expression level, MMC induced depletion of GCN2 in rats. Interestingly, GCN2 loss has been reported to promote oxidative stress and inflammatory-mediated DNA damage [58], a proposed pathogenic setup for PAH development. GCN2, encoded by the gene eukaryotic translation initiation factor 2α kinase 4 (*EIF2AK4*), is biallelically inactivated in familial PVOD/PCH, which is inherited as an autosomal recessive trait [59,60]. Based on these observations, it can be hypothesized that MMC, an alkylating agent, can directly trigger pulmonary vascular dysfunction via DNA damage signaling, and that a similar mechanism may underlie familial PVOD/PCH with biallelic inactivation of *EIF2AK4* (Figure 2).

## 5. DNA Repair Pathways and Cell Cycle Checkpoints in PAH

Despite the relatively high frequency of chromosomal abnormalities in PAH PAECs, clonal analysis of endothelial colony-forming cells from these cultures showed that the genome remained grossly stable for up to 15 passages [61]. This suggests there is no major defect in DNA repair, and indeed it may even be enhanced in PAH cells. As we review below, most studies of DNA repair to date have been performed in PASMCs, with relatively little information known about PAECs. However, the consensus thus far suggests opposite effects in these two cell types, with evidence of decreased repair in PAH-PAECs, but increased repair in PASMCs.

Peroxisome proliferator-activated receptor γ (PPAR-γ) is a nuclear receptor known to regulate fatty acid storage and glucose metabolism. It has been implicated in multiple diseases, including cancer, obesity, diabetes, and cardiovascular diseases, particularly in ECs [62]. Mice with EC-specific deletion of PPAR-γ develop PH under hypoxia, which is persistent after re-oxygenation [63]. In PAECs and PASMCs, PPAR-γ promotes cell survival and suppresses proliferation via interaction with Apelin [64]. Using an unbiased proteomics approach, Li and colleagues showed that PPAR-γ interacts with MRN (MRE-11-RAD50-NBS1), promoting ATM signaling, and is also essential for UBR5 activity targeting ATM interactor (ATMIN) [65]. Hence, upon DNA damage, ATMIN is degraded by UBR5, leading to ATM activation. However, this axis is dysfunctional in PAH-PAECs, with reduced PPAR-γ-UBR5 interaction, elevated ATMIN which leads to progressive DNA damage, and impaired repair in these cells (Figure 2). Consistent with the in vitro pathway findings, the results were validated in PAH-PAECs and PAH-lung tissues [65]. Furthermore, the reduction of ATMIN in PAH-PAECs reduced synthetic DNA damage comparable to the control cells. Overall, the study established a non-canonical pathway for DDR and DNA repair via PPAR-γ suggesting the importance of PPAR-γ in EC homeostasis and maintenance of genome integrity (Figure 2).

**Figure 3:**
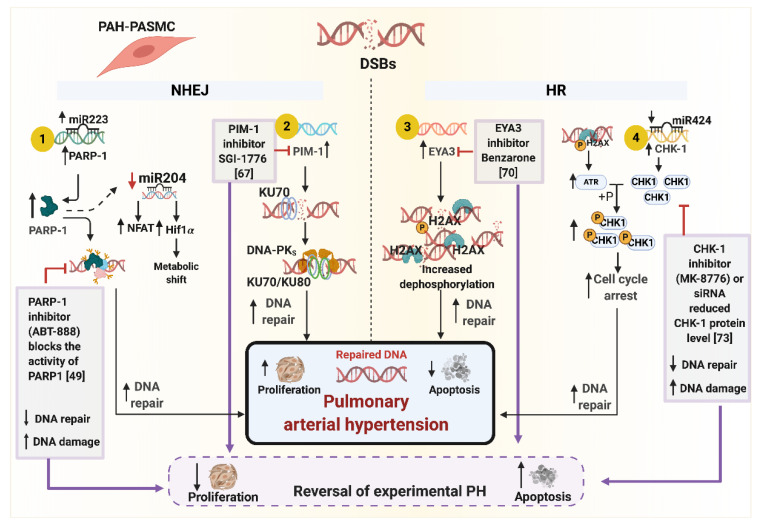
Dysregulated DNA damage response genes involved in pro-proliferative and apoptosis resistance characteristics of PAH-PASMCs. The figure represents the DDR genes and their downstream pathways implicated in PAH pathogenesis (1) PARP-1, (2) PIM-1, (3) EYA3, and (4) CHK1. These genes are upregulated in PAH-PASMCs leading to increased DNA repair and proliferation and reduced apoptosis. Studies have shown specific pharmacological inhibitors like ABT-888 (PARP1 inhibitor), SGI-1776 (PIM1 inhibitor), Benzarone (EYA3 inhibitor), and MK-8776 (CHK1 inhibitor) downregulate their target genes, leading to reduced proliferation and increased apoptosis, suggesting their therapeutic potential in PAH. DSBs, Double-stranded breaks; NHEJ, Non-Homologous End Joining; HR, Homologous Recombination; DSBs, Double-strand breaks; PARP1, Poly (ADP-Ribose) Polymerase 1; PIM1, Proto-Oncogene Serine/Threonine Kinase; EYA-3, Eyes Absent Homolog 3; CHK-1, Checkpoint Kinase 1; KU70, X-Ray Repair Cross Complementing 6; KU80, X-Ray Repair Cross Complementing 5; NFAT, Nuclear Factor of Activated T-Cells; HIF1α, Hypoxia-Inducible Factor 1-α; H2AX, H2a Histone Family Member X; P, phosphoryl group; ATR, Ataxia Telangiectasia and Rad3-Related Protein; miR223, microRNA 223; miR204, microRNA 204; miR424, microRNA 223; DNA-PKs, DNA-Dependent Protein Kinases; PAH, Pulmonary Arterial Hypertension. The numbers represented in square brackets cite the reference for that study. Created with BioRender.com.

One of the first responder proteins to detect DNA damage is Poly [ADP-ribose] polymerase 1 (PARP1). Meloche and colleagues first reported decreased microRNA miR-223, increased PARP-1 expression and associated proliferation/apoptosis imbalance in PAH [66] (Figure 3). Expectedly, treatment with PARP-1 inhibitor, ABT-888, induced more DNA damage in PASMCs; however, it also initiated anti-proliferative and pro-apoptotic signaling via reversal of miR-204-dependent NFAT and Hif1-α levels (Figure 3) [49]. These observations were recapitulated in the in vivo experimental PAH setup where treatment with ABT-888 reversed the effects of monocrotaline (MCT) and Sugen-induced PAH, as represented by reduced PA pressure and RV hypertrophy. Overall, the study was the first to report the augmented DNA damage response pathway in PAH via PARP-1 activation. Based on the in vivo results, PARP-1 inactivation was proposed as a potential therapeutic marker. However, the myriad effects of PARP-1 in the regulation of stress-regulated cell signaling along with other potential DNA damage inducers like tumor necrosis factor-α (TNF-α) and interleukin-6 (IL-6) cannot be ruled out. The immediate effect of PARP-1 inhibitor on increased DNA damage may route cells towards a far more unfavorable cellular environment, encouraging a broader exploration of multifaceted PARP-1 in PAH pathogenesis.

In another study from the same group, Lampron and colleagues explored the role of Moloney murine leukemia provirus integration site (PIM1) [67], a regulator of NHEJ repair pathway gene in PAH [68]. Increased PIM1 expression was observed in PAH lungs and PASMCs as compared to the controls [67]. PIM1 inhibition itself did not increase DNA damage; however, it reduced the expression of its downstream target, KU70, encoded by the gene X-Ray Repair Cross Complementing 6 (*XRCC6*) that stabilizes the ends of DSBs. Overall, PIM1 reduction significantly reduced the DNA repair capacity of the cells by inhibiting the primary events involved in DNA repair. Treatment with PIM1 inhibitors SGI-1776 and TP-3654 affected proliferation and apoptosis in PAH-PASMCs in an anti-pathological manner (Figure 3) [67]. These observations were recapitulated in PH animal models, where pharmacological intervention in two experimental rat models with PIM1 inhibitors improved the hemodynamic characteristics, reduced vascular remodeling, reduced proliferation, and enhanced apoptosis [67]. As opposed to the previous pharmacological inhibitor of the DNA repair pathway studied by this group [66], PIM1 inhibition does not expose vascular cells to additional genetic insults.

The majority of the studies focused on H2AX to quantify the repair of DSBs. H2AX is the central player that binds to the DSBs, and the status of phosphorylation and dephosphorylation of H2AX governs the assembly of DDR complexes [69]. The phosphorylation of H2AX at Tyrosine-142 (Y142) residue is constitutive. Under a DNA-damaging environment, H2AX is phosphorylated at Serine-139 (S139) residue, allowing cells to initiate the DNA damage response. To initiate the repair machinery, EYA3, a tyrosine phosphatase, dephosphorylates the Y142 residue, permitting the assembly of repair complexes at the site of DNA damage. If Y142 is not de-phosphorylated, the cell initiates apoptosis [69]. In this context, Wang et al., reported increased levels of EYA3 protein in PAH-PASMCs and distal pulmonary arteries, suggesting an elevated repair mechanism in PAH pathogenesis (Figure 3) [70]. They also showed that in the presence of hydrogen peroxide, EYA3 provides a protective mechanism aiding in PASMC survival which was completely reversed in a setting with attenuated EYA3 expression. The treatment of cells with the small molecule inhibitor, benzarone, established the relationship between EYA3 tyrosine phosphatase activity and cell survival under a DNA damaging environment (Figure 3) [70]. Inhibition of EYA3 tyrosine phosphatase activity in Sugen-hypoxia rats improved pulmonary hemodynamics and vascular remodeling [70]. Using genetically modified mice with an inactive EYA3 (Eya3^D262N^), the authors showed that under hypoxic conditions, Eya3^D262N^ mice are protected from developing PH as opposed to the control mice under similar conditions [70].

In cancerous cells, checkpoint kinase-1 (CHK-1) acts as a nexus for cell survival under DNA damaging conditions by halting cell progression and triggering DNA repair [71,72]. Bourgeois and colleagues reported increased levels of checkpoint kinase-1 (CHK-1) in PAH-PASMCs and distal pulmonary arteries but not in PAH-PAECs (Figure 3) [73]. This expression correlated with the increased DNA damage markers, γH2AX and RPA32. Further, in PAH-PASMCs, the direct upstream activator of CHK-1, phospho-ATK [74,75], was upregulated, and mir-424, which targets CHK-1 [76], was also found to be downregulated. Upon treatment with DNA-damage-inducing compounds, an increase in CHK-1 levels was observed with anti-proliferative and pro-apoptosis characteristics (Figure 3) [73]. To understand the pharmacological effects, treatment of PAH-PASMCs with the CHK1 kinase inhibitor, MK-8776, exacerbated the DNA damage while controlling the proliferation and enhancing apoptosis. The results also validated with small interfering RNA (siRNA) revealed enhanced expression of CHK-1 upregulates RAD-51 [73]. This contrasts with the findings in PAECs in a separate study, where BMPR2-deficient PAECs showed a reduced level of RAD51 and lung tissue from IPAH patients had attenuated RAD51 levels [77]. In vivo studies supported the therapeutic potential of the CHK-1 inhibitor by reducing the hemodynamic parameters associated with increased DNA damage in fawn-hooded rats with already developed PAH; however, no reduction in RV hypertrophy was observed [73]. In a separate experiment on MCT rats, pharmacological inhibition of CHK-1 using MK-8776 revealed marginal improvement in hemodynamic parameters and enhanced reduction in vascular remodeling [73]. Overall, the study suggests that ubiquitous expression of CHK-1 allows the vascular cells to thrive under excessive DNA damaging conditions by providing a survival advantage [78].

## 6. DNA Damage and Mitochondria

Mitochondria serve an indispensable role in cellular health under various stresses. They have their own genome, a circular DNA molecule (mtDNA) of approximately 16,500 base pairs. Despite the higher levels of ROS, mtDNA repair mechanisms are reduced compared with the nucleus, increasing the likelihood of mtDNA damage. Several studies have reported abnormalities of mitochondrial function in PAH pathogenesis, including increased glycolytic metabolism, decreased mitochondrial copy number, and enhanced fission [79,80,81,82,83]. PARP1 plays a role in regulating mitochondrial energy metabolism and may therefore contribute to the alterations seen in PAH cells [84,85]. However, mitochondrial function is a very broad topic that has been extensively reviewed by others [83,86], and thus here we focus on the studies that directly relate to mitochondrial DNA damage.

**Figure 4:**
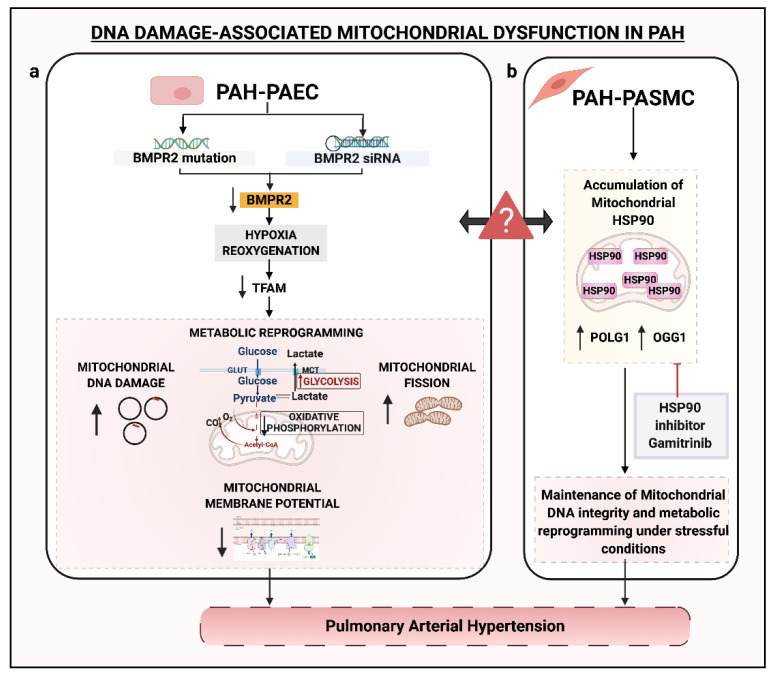
DNA damage-associated mitochondrial dysfunction in (**a**) PAH-PAEC and (**b**) PAH-PASMC. (**a**) In PAH-PAECs, reduced expression of BMPR2 accompanied by hypoxia-reoxygenation is associated with reduced expression of the mitochondrial DNA maintenance gene, TFAM, increased mitochondrial DNA damage, and decreased mitochondrial membrane potential. These factors, associated with reduced oxidative phosphorylation and increased glycolysis, trigger endothelial dysfunction in PAH-PAECs [78]. (**b**) In PAH-PASMCs, increased mitochondrial HSP90 accumulation along with an increased expression of POLG1 and OGG1 is proposed as a regulatory mechanism in the maintenance of mitochondrial DNA and metabolic reprogramming under stressful conditions [88]. The question mark symbol between the two cell types indicates a lack of studies showing crosstalk between the two cell types. DNA, Deoxyribonucleic acid; TFAM, Transcription Factor A Mitochondrial; GLUT, Glucose transporter; MCT, monocarboxylate transporter; O_2_, Oxygen; CO_2_, Carbon dioxide; HSP90, Heat shock protein 90; POLG1, Mitochondrial DNA polymerase γ; OGG1, 8-Oxoguanine glycosylase. Created with BioRender.com.

Diebold and colleagues studied the role of BMPR2 signaling in mitochondrial DNA (mtDNA) damage and metabolism in apoptosis of PAECs in PAH [78]. Using human PAECs with siRNA-downregulated BMPR2 and PAH-PAECs with inherent *BMPR2* mutations, it was revealed that BMPR2 dysregulation can promote mtDNA damage when exposed to reoxygenation. Hypoxia-reoxygenation leads to reduced expression of TFAM and mitofusin 1 and 2 proteins. While TFAM is involved in mtDNA maintenance and repair, mitofusin 1 and 2 regulate mitochondrial fission. Consistent with their functions, enhanced accumulation of a 4977-bp mtDNA deletion and increased mitochondrial fission was observed in BMPR2-depleted PAECs [78] (Figure 4). This suggested that hypoxia-reoxygenation was a severe pathogenic trigger linked to the mitochondrial genetic abnormality. Overall, these studies imply that the genomic instability in PAH vasculature is not only confined to the nuclear genome but also impacts the mtDNA, which is far more susceptible to damage as compared to the nuclear genome [87].

Boucherat et al. showed an important role of stress responder chaperone HSP90 in the maintenance of mitochondrial function [88]. Under a pathogenic PAH environment, HSP90 favorably localized within the mitochondria of PAH-PASMCs to preserve the mtDNA and its bioenergetic functions (Figure 4) [88]. Contrary to that, the cytosolic HSP90 displayed no conclusive role in PAH pathogenesis. On the same lines, in vitro and in vivo studies established a potential therapeutic role of Gamitrinib, a selective inhibitor of mtHSP90, in repressing PAH-PASMCs proliferation and initiating apoptosis, and reversing experimental PAH in MCT treated rats [88]. Within PAH-PASMCs, Gamitrinib reduced the endogenous overexpression of mtDNA maintenance genes, DNA polymerase γ (*POLG1*) and *OGG1* (Figure 4) [88]. OGG1, involved in BER, is pertinent to the elimination of oxidative DNA damage. Along with its role in mitochondrial maintenance, overexpression of OGG1 might implicate higher oxidative stress-induced damage in mtDNA, although this needs further validation.

## 7. BMPR2 and DNA Damage

Using a meta-analysis approach, Li and colleagues analyzed the expression profiles of IPAH (PASMCs and whole lung) and HPAH patients with *BMPR2* genetic variants (PAECs) to reveal 586 up-regulated and 372 down-regulated genes in PAH [89]. In silico analyses revealed significant enrichment of chromatin organization genes, predominantly regulated by the transcription factors, SP1 and NKX3. This also included over 35 genes involved in DNA repair. More importantly, both SP1 and NKX3 are well-known regulators of DSB repair. This observation suggests that the multifactorial channeling of cell signaling in PAH vasculopathy could be initiated by genomic instability. Similar to human expression profiles, an enrichment of DNA repair genes was also reported in rats in the setting of PAH. The authors went on to understand the relationship between two known hallmarks of PAH pathogenesis: altered BMP signaling and DNA damage. Using discrete in vitro methods, the authors established that MMC-induced DNA damage significantly downregulates BMPR2 expression and that BMPR2 is critical for DNA damage control in ECs [89]. However, these observations were confined to ECs; PASMCs showed no such correlation. Also, a transcription binding site for BRCA1, an important gene in cancer pathogenesis, was revealed using the ChIP assay and established a regulatory feed-back loop mechanism for *BMPR2* and *BRCA1*. Over the years, several theories and relevant studies have suggested a cancer-like pathogenesis in PAH. Establishing the interaction between BMPR2 and BRCA1 strengthened the link between cancer and PAH; however, the findings need to be corroborated with a larger dataset.

Consistent with the downregulation of BMPR2 under a DNA-damaging environment, Vattulainen-Collanus and colleagues uncovered a previously unknown role of BMP signaling in the protection of cells from genomic insult [77]. They showed that MMC treatment reduced the expression of BMPR2, BRCA1, and RAD51. Also, siRNA against BMPR2 reduced BRCA1 and RAD51 transcript and protein levels by over 50% and also lead to a 61% higher level of fragmented DNA. Further, activation of BMPR2 signaling by BMP9 partially rescues RAD51 and reduces sensitivity to DNA damage agents. At the epigenetic level, miR-96 is known to target the coding region of RAD51 and downregulate its expression [90]. Vattulainen-Collanus and colleagues hypothesized that repressing miR-96 via BMP9–BMPR2 signaling was critical for RAD51 expression and maintained pulmonary microvascular ECs homeostasis via managing DNA repair (Figure 2). To validate the in vitro results, the role of RAD-51 was studied in *BMPR2*^R899X/+^ mice, which develop mild PH beyond 6 months [91]. It was observed that RAD51 expression was repressed in PMVECs and PASMCs extracted from *BMPR2*^R899X/+^ mice as compared to control mice, suggesting a regulatory link between BMPR2 and RAD51 [77]. Similarly, lung sections from IPAH patients had a significant reduction of RAD51 in the endothelium of pulmonary arteries compared to control samples.

## 8. Conclusions and Future Directions

Over the last two decades, considerable progress has been made in understanding the pathobiology of PAH, with both genetic and environmental factors playing crucial roles in disease pathogenesis. It was very recently that the DNA damage and DDR malfunction was recognized. Several studies have reported persistent DNA damage (nuclear and mitochondrial) in the vascular cells across different types of PAH. Intriguingly, the increased DNA damage in blood cells of PAH patients and their first-degree relatives suggests that it may be a genetically-determined susceptibility factor that pre-dates disease initiation [46]. The increased incidence of PVOD following chemotherapy with MMC also suggests DNA damage as an early driver of pulmonary vascular remodeling [57]. Importantly, however, the types of DNA damage most prevalent in PAH and their cause(s) remain to be fully determined. Some studies suggest that the downregulation of BMPR2 may play a pivotal role in DNA damage [77,89], yet the incidence of DNA damage measured by micronucleus assay and γH2AX staining trended lower in HPAH cells compared with APAH-CHD [46], emphasizing that gaps in knowledge remain.

Beyond DNA damage, various studies focused on the status of DDR in PAH have ascertained a dysfunctional repair mechanism leading to an augmented apoptosis-resistant and proliferative phenotype [49,67,73,88]. Taken together, it is likely that the pathways underlying DDR play a key role in PAH. DDR involves a complex network of proteins that recognize different types of DNA damage and orchestrate the repair process. At present, there is not enough evidence to draw conclusions on the status of different DNA repair pathways in PAH. There are reports suggesting amplified DDR in PAH-PASMCs [49,67], while a few stand in contradiction (Table 1: OGG1, RAD51), reporting reduced DDR in PAH-PAECs [47,65,89] under different DNA damaging environments (Table 1). One of the major reasons behind this gap is a lack of paired cell types from the patients in the same study, as well as replication of the findings across different types of PAH. The absence of information on the underlying genetic mutations and polymorphisms that may act as genetic modifiers is an additional limitation, but this can be addressed fairly readily by more extensive use of whole-genome sequencing. PAH is a complex disease, and in this framework, it is important to get a comprehensive view of each variable of the disease to make significant progress.

The similarity of PAH to a quasi-neoplastic phenotype has triggered the study of DDR inhibitors as a potential therapeutic approach. As outlined in Figure 3, specific pharmacological inhibitors like ABT-888 (PARP1 inhibitor), SGI-1776 (PIM1 inhibitor), Benzarone (EYA3 inhibitor), and MK-8776 (CHK1 inhibitor) downregulate their target genes, leading to reduced proliferation and increased apoptosis, suggesting their therapeutic potential in PAH [49,67,70,73]. However, the cancer-like mechanism has a broad spectrum of phenotypes delineated by different stages of the disease, organs, and cell types. DDR inhibitors have shown some success, but the additional damage and associated detrimental effects on health across different vascular compartments cannot be ignored [49,67,70,73]. It is important to note that, by necessity, the majority of these studies are performed on PAH-vascular cells, PAECs and PASMCs, derived from end-stage tissues, since such cells cannot be obtained at earlier stages of the disease [39,40,42,44,45,46,47,48,49,61,66,67,70,73,77,78,88,89]. The dysfunctional DDR or augmented DNA damage could be the result of surrounding variables like inflammation, hypoxia etc. Efforts need to be made to understand the cellular microenvironment and the transition of cells from an adaptive to maladaptive DDR. A prospective therapeutic can only be developed once a delineation between the adaptive and maladaptive switch has been established.

PAH goes undiagnosed until the later stages of the disease. This imposes a major limitation to define the early diagnosis markers. The lung tissue samples are only available once the patient undergoes a transplant. In such a scenario, it is difficult to find biomarkers for early disease prediction. Additional reports on the first-degree relatives and in blood cells from PAH patients may assist in establishing novel prognosis markers along with validation of DNA damage and ROS levels. Different cell types in PAH have different pathogenic presentations, and the lack of data on more than one cell type emerges as another major limitation across most of these studies. Further, the majority of studies have explored DNA damage and repair pathways only in Group 1 PAH. Studying these mechanisms in other groups of PH will be important to understand the similarities and differences in molecular mechanisms. Thought should be given to innovative methods to get access to patient cells and tissues at different developmental stages. If not the lung, are there other tissues that can mirror the lung phenotype? In this context, the use of iPSCs and 3D printing of organs with known hits for disease pathogenesis can be explored. To produce significant improvements in patient outcomes, priority should be given to the development of comprehensive functional-based predictive biomarker assays. With current advancements, techniques like single-cell transcriptional and translational studies will give a better understanding of the individual pathogenic (or pro-pathogenic) events in a cell in context to its microenvironment. These studies, in combination with high-throughput sequencing to underscore genetic and epigenetic changes, will supplement the exploration of the pathological mechanism at the systemic and lung-specific level.

## Figures and Tables

**Table 1:** Genes associated with the DNA damage and response pathways in pulmonary arterial hypertension.

DNA Damage and Response Genes	PAH-PAECs	PAH-PASMCs
Base Excision Repair		
*OGG1* (8-Oxoguanine DNA Glycosylase)	Not known	Increased expression [88]
		Reduced expression ** [67]
Homologous recombination		
*RAD51* (RAD51 Recombinase)	Reduced expression [77]	Increased expression [73]
*BRCA1* (Breast and Ovarian Cancer Susceptibility Protein 1)	Reduced expression ^#^ [77]	Reduced expression [89]
*NBS1* (Nibrin)	Not known	Reduced expression ***
Non-homologous end-joining		
*XRCC6* (Ku70) (X-Ray Repair Cross Complementing 6)	Not known	Reduced expression **
*PARP-1* (Poly (ADP-Ribose) Polymerase 1)	Not known	Increased expression [49]
Other genes involved in regulation of DNA damage		
*BMPR2*	Reduced expression ^#^ [89]	No change in expression * [89]
*TFAM*	Reduced expression [78]	Not known
*TOPBP1* (DNA Topoisomerase II Binding Protein 1)	Reduced expression [48]	Not known
*PPARG-UBR5*	No change in expression but reduced interaction observed [65]	Not known
*ATMIN*	Increased expression [65]	Not known
*PIM1*	Not known	Increased expression [67]
*EYA3*	Not known	Increased expression [70]
*CHK1* (Check point Kinase-1)	No association [73]	Increased expression [73]

PAH, Pulmonary arterial hypertension; PAECs, Pulmonary artery endothelial cells; PASMCs, Pulmonary artery smooth muscle cells; ^#^, Experiment performed in control PAECs with MMC treatment; *, Experiment performed in control PASMCs with MMC treatment; **, Experiment performed in control PASMCs with *PIM-1* inhibition; *** Experiment performed in control PAH-PASMCs with siRNA for *FOXM1*.
